# Supplementary figures and images for: High laboratory mouse pre-weaning mortality associated with litter overlap, advanced dam age, small and large litters
Source: PLoS One. 2020 Aug 12;15(8):e0236290. doi: 10.1371/journal.pone.0236290 (PMC7423063; doi:10.1371/journal.pone.0236290)

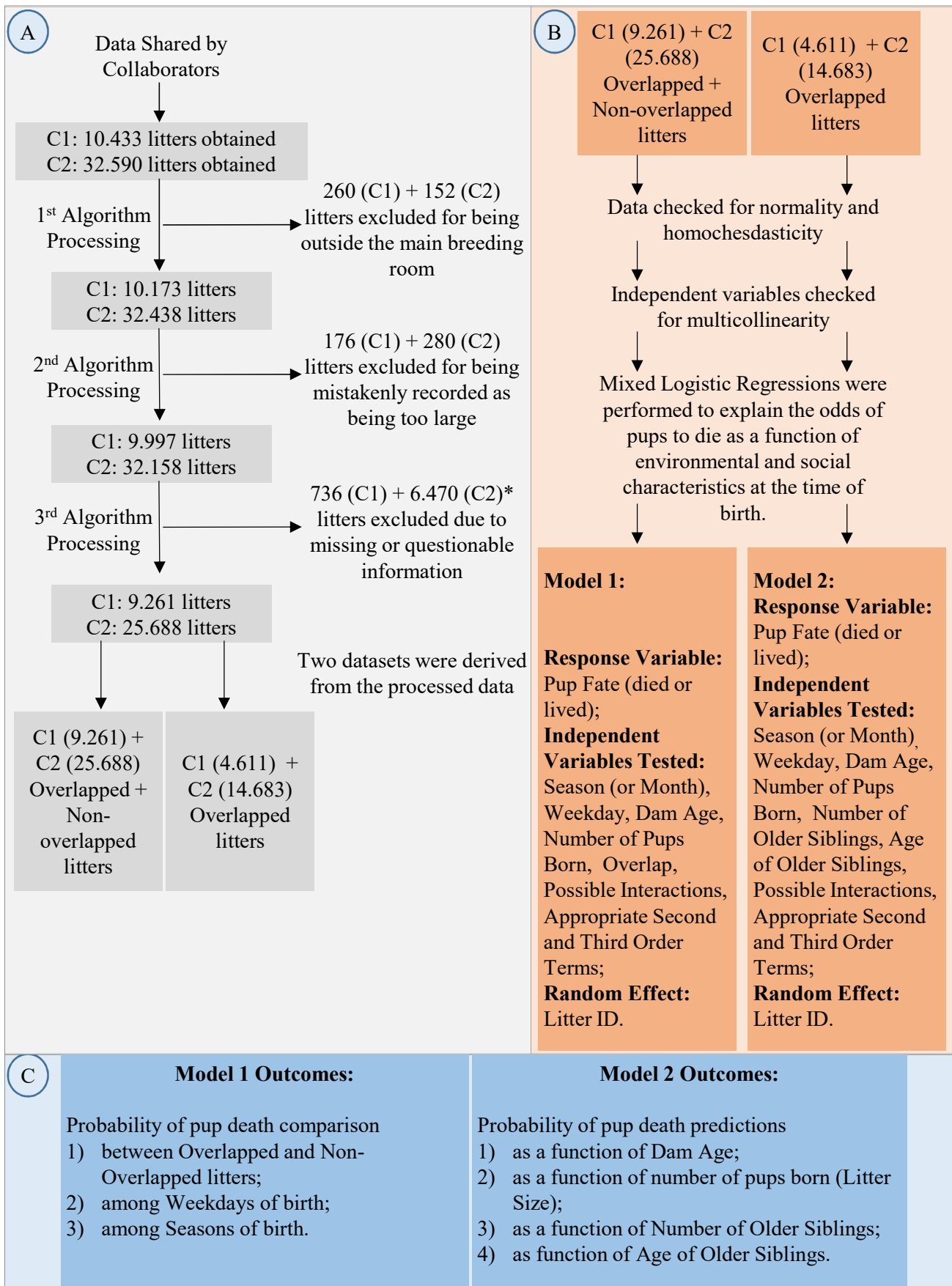

Supplement: S1 Fig — Schematic illustration of the data analysis process performed on the data provided by each of the two collaborators. (A) After original datasets were processed with the help of a data-cleaning algorithm, the data were split into two datasets: One with overlapped and non-overlapped litters and another with only overlapped litters. (B) Model 1 was performed to evaluate which environmental and social factors were a risk for pup mortality, while Model 2 was performed to provide in more details how the social factors affect pup mortality in overlapped litters. Model outcomes are listed in (C). (PDF) [file pone.0236290.s001.pdf]

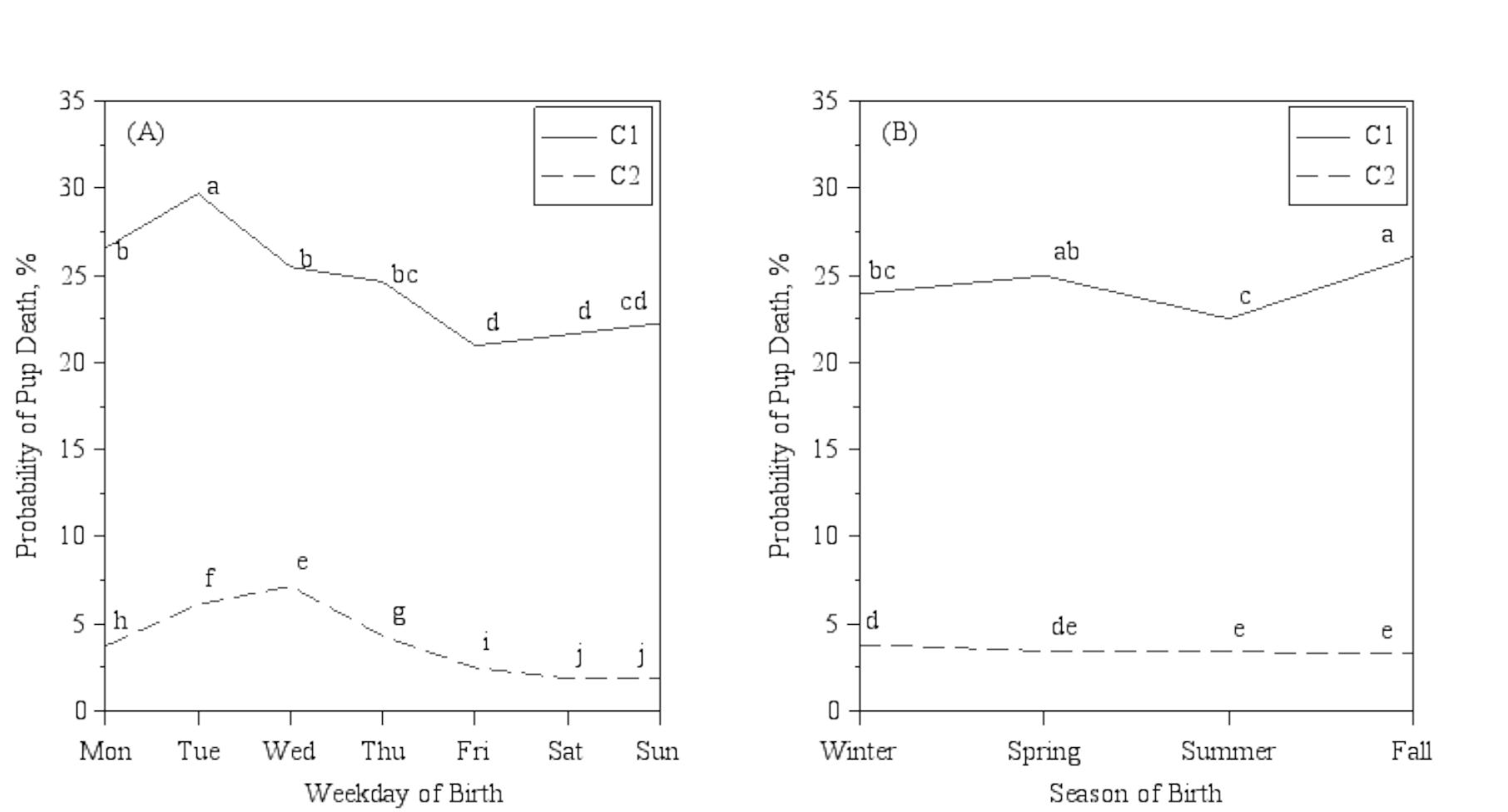

Supplement: S2 Fig — Predicted Probability (least-square means) of a pup to die as a function of (A) Weekday and (B) Season of birth at C1, the Babraham Institute, and C2, the Wellcome Sanger Institute. Data points with distinct labeled letters indicate statistical difference at 95% confidence level. (TIF) [file pone.0236290.s002.tif]

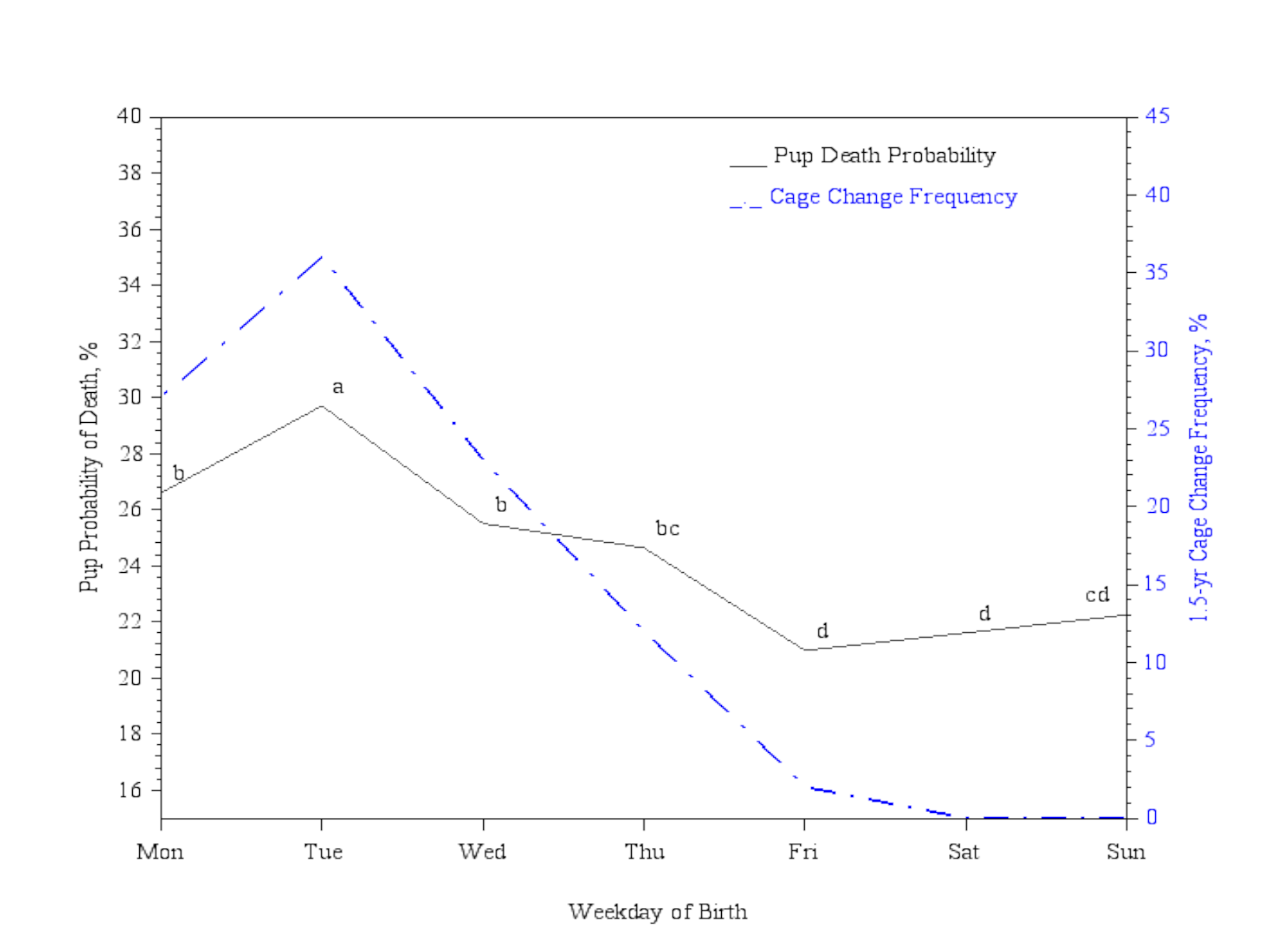

Supplement: S3 Fig — Cage change frequency and predicted probability (least-square means) of a pup to die as a function of Weekday at C1, the Babraham Institute. Data points with distinct labeled letters indicate statistical difference at 95% confidence level. Cage change frequency is depicted as the percentage per weekday of the 78 cage change episodes which happened from April 2018 to November 2019 (available data records), in the studied room of C1. (TIF) [file pone.0236290.s003.tif]
