# Supplementary material for: High laboratory mouse pre-weaning mortality associated with litter overlap, advanced dam age, small and large litters
Source: PLoS One. 2020 Aug 12;15(8):e0236290. doi: 10.1371/journal.pone.0236290 (PMC7423063; doi:10.1371/journal.pone.0236290)
Supplement: S1 Table — n.a. = not applicable. aVariable Litter Size was centered by its mean. (PDF) [file pone.0236290.s004.pdf]

Effect	Estimate	Standard Error	t Value	Pr > |t|
**Intercept**	-1.0546	0.0751	-14.04	<0.0001
**Collaborator C1**	2.3067	0.1011	22.81	<0.0001
**Collaborator C2**	0.0000	n.a.	n.a.	n.a.
**Season Fall**	-0.1468	0.0354	-4.14	<0.0001
**Season Spring**	-0.1082	0.0360	-3.00	0.0027
**Season Summer**	-0.1234	0.0400	-3.09	0.0020
**Season Winter**	0.0000	n.a.	n.a.	n.a.
**Weekday 1**	-0.0054	0.0517	-0.11	0.9162
**Weekday 2**	0.7230	0.0472	15.33	<0.0001
**Weekday 3**	1.2433	0.0463	26.86	<0.0001
**Weekday 4**	1.4133	0.0453	31.18	<0.0001
**Weekday 5**	0.8705	0.0461	16.88	<0.0001
**Weekday 6**	0.3000	0.0482	6.22	<0.0001
**Weekday 7**	0.0000	n.a.	n.a.	n.a.
**Dam Age**	0.0046	0.0003	15.89	<0.0001
**Litter Size**[a]	-0.7104	0.0134	-53.20	<0.0001
**Litter Size^2**[a]	0.0345	0.0009	36.73	<0.0001
**Overlap No**	-0.4969	0.0240	-20.67	<0.0001
**Overlap Yes**	0.0000	n.a.	n.a.	n.a.
**Collaborator C1* Season Fall**	0.2607	0.0488	5.35	<0.0001
**Collaborator C1* Season Spring**	0.1647	0.0495	3.33	0.0009
**Collaborator C1* Season Summer**	0.0435	0.0541	0.80	0.4210
**Collaborator C1* Season Winter**	0.0000	n.a.	n.a.	n.a.
**Collaborator C2* Season Fall**	0.0000	n.a.	n.a.	n.a.
**Collaborator C2* Season Spring**	0.0000	n.a.	n.a.	n.a.
**Collaborator C2* Season Summer**	0.0000	n.a.	n.a.	n.a.
**Collaborator C2* Season Winter**	0.0000	n.a.	n.a.	n.a.
**Collaborator C1* Weekday Sunday**	0.04295	0.0683	0.63	0.5292
**Collaborator C1* Weekday Monday**	-0.4495	0.0639	-7.04	<0.0001
**Collaborator C1* Weekday Tuesday**	-0.8161	0.0637	-12.81	<0.0001
**Collaborator C1* Weekday Wednesday**	-1.1965	0.0626	-19.11	<0.0001
**Collaborator C1* Weekday Thursday**	-0.6996	0.0632	-11.06	<0.0001
**Collaborator C1* Weekday Friday**	-0.3375	0.0648	-5.21	<0.0001
**Collaborator C1* Weekday Saturday**	0.0000	n.a.	n.a.	n.a.
**Collaborator C2* Weekday Sunday**	0.0000	n.a.	n.a.	n.a.
**Collaborator C2* Weekday Monday**	0.0000	n.a.	n.a.	n.a.
**Collaborator C2* Weekday Tuesday**	0.0000	n.a.	n.a.	n.a.
**Collaborator C2* Weekday Wednesday**	0.0000	n.a.	n.a.	n.a.
**Collaborator C2* Weekday Thursday**	0.0000	n.a.	n.a.	n.a.
**Collaborator C2* Weekday Friday**	0.0000	n.a.	n.a.	n.a.
**Collaborator C2* Weekday Saturday**	0.0000	n.a.	n.a.	n.a.
**Collaborator C1*Dam Age**	-0.0013	0.0003	-3.81	0.0001
**Collaborator C2*Dam Age**	0.0000	n.a.	n.a.	n.a.
**Collaborator C1*Litter Size**[a]	0.0484	0.0066	7.37	<0.0001
**Collaborator C2*Litter Size**[a]	0.0000	n.a.	n.a.	n.a.
**Collaborator C1*Overlap No**	0.1426	0.0332	4.30	<0.0001
**Collaborator C1*Overlap Yes**	0.0000	n.a.	n.a.	n.a.
**Collaborator C2*Overlap No**	0.0000	n.a.	n.a.	n.a.
**Collaborator C2*Overlap Yes**	0.0000	n.a.	n.a.	n.a.

Type III (Partial) Tests of Fixed Effects			
**Effect**	**Num DF**	**F Value**	**Pr > F**
**Collaborator**	1	579.79	<0.0001
**Season**	3	5.84	0.0006
**Weekday**	6	256.88	<0.0001
**Dam Age**	1	511.62	<0.0001
**Litter Size**[a]	1	2563.59	<0.0001
**Litter Size^2**[a]	1	1349.04	<0.0001
**Overlap**	1	658.04	<0.0001
**Collaborator*Season**	3	13.55	<0.0001
**Collaborator*Weekday**	6	102.88	<0.0001
**Collaborator*Dam Age**	1	14.48	0.0001
**Collaborator*Litter Size**[a]	1	54.30	<0.0001
**Collaborator*Overlap**	1	18.46	<0.0001

n.a.=not applicable.

[a]Variable Litter Size was centered by its mean.
