# Supplementary material for: High laboratory mouse pre-weaning mortality associated with litter overlap, advanced dam age, small and large litters
Source: PLoS One. 2020 Aug 12;15(8):e0236290. doi: 10.1371/journal.pone.0236290 (PMC7423063; doi:10.1371/journal.pone.0236290)
Supplement: S2 Table — n.a. = not applicable. aVariable Litter Size was centered by its mean. (PDF) [file pone.0236290.s005.pdf]

Effect	Estimate	Standard Error	t Value	Pr > |t|
**Intercept**	-11.5390	0.3921	-29.43	<0.0001
**Collaborator C1**	-2.2587	0.5681	-3.98	<0.0001
**Collaborator C2**	0.0000	n.a.	n.a.	n.a.
**Weekday 1**	-0.0671	0.2256	-0.30	0.7663
**Weekday 2**	0.5713	0.2105	2.71	0.0066
**Weekday 3**	1.1023	0.2139	5.15	<0.0001
**Weekday 4**	1.5854	0.2116	7.49	<0.0001
**Weekday 5**	0.3004	0.2109	1.42	0.1544
**Weekday 6**	0.0120	0.2184	0.05	0.9563
**Weekday 7**	0.0000	n.a.	n.a.	n.a.
**Season Fall**	0.0219	0.1584	0.14	0.8902
**Season Spring**	0.4246	0.1562	2.72	0.0066
**Season Summer**	0.4826	0.1564	3.09	0.0020
**Season Winter**	0.0000	n.a.	n.a.	n.a.
**Dam Age**	0.0083	0.0012	7.25	<0.0001
**Litter Size**[a]	-0.2233	0.0228	-9.78	<0.0001
**Litter Size^2**[a]	0.0814	0.0055	14.80	<0.0001
**Sibling Number**	0.02873	0.0396	0.73	0.4676
**Sibling Age**	-0.0143	0.0194	-0.74	0.4618
**Sibling Number*Sibling Age**	0.0181	0.0029	6.29	<0.0001
**Collaborator C1*Sibling Age**	0.5338	0.0217	24.61	<0.0001
**Collaborator C2*Sibling Age**	0.0000	n.a.	n.a.	n.a.
**Collaborator C1*Sibling Number**	0.4587	0.0464	9.88	<0.0001
**Collaborator C2*Sibling Number**	0.0000	n.a.	n.a.	n.a.
**Collaborator C1*Litter Size**[a]	0.2170	0.0502	4.33	<0.0001
**Collaborator C2*Litter Size**[a]	0.0000	n.a.	n.a.	n.a.
**Collaborator C1* Season Fall**	0.5386	0.3721	1.45	0.1478
**Collaborator C1* Season Spring**	-0.420	0.3566	-0.12	0.9063
**Collaborator C1* Season Summer**	-0.7081	0.3697	-1.92	0.0554
**Collaborator C1* Season Winter**	0.0000	n.a.	n.a.	n.a.
**Collaborator C2* Season Fall**	0.0000	n.a.	n.a.	n.a.
**Collaborator C2* Season Spring**	0.0000	n.a.	n.a.	n.a.
**Collaborator C2* Season Summer**	0.0000	n.a.	n.a.	n.a.
**Collaborator C2* Season Winter**	0.0000	n.a.	n.a.	n.a.
**Collaborator C1* Weekday Sunday**	0.9696	0.5306	1.83	0.0676
**Collaborator C1* Weekday Monday**	-0.7351	0.4891	-1.50	0.1328
**Collaborator C1* Weekday Tuesday**	-0.4743	0.5124	-0.93	0.3546
**Collaborator C1* Weekday Wednesday**	-2.0820	0.4996	-4.17	<0.0001
**Collaborator C1* Weekday Thursday**	-0.6480	0.5063	-1.28	0.2006
**Collaborator C1* Weekday Friday**	-0.0665	0.5011	-0.13	0.8944
**Collaborator C1* Weekday Saturday**	0.0000	n.a.	n.a.	n.a.
**Collaborator C2* Weekday Sunday**	0.0000	n.a.	n.a.	n.a.
**Collaborator C2* Weekday Monday**	0.0000	n.a.	n.a.	n.a.
**Collaborator C2* Weekday Tuesday**	0.0000	n.a.	n.a.	n.a.
**Collaborator C2* Weekday Wednesday**	0.0000	n.a.	n.a.	n.a.
**Collaborator C2* Weekday Thursday**	0.0000	n.a.	n.a.	n.a.
**Collaborator C2* Weekday Friday**	0.0000	n.a.	n.a.	n.a.
**Collaborator C2* Weekday Saturday**	0.0000	n.a.	n.a.	n.a.

Type III (Partial) Tests of Fixed Effects			
**Effect**	**Num DF**	**F Value**	**Pr > F**
**Collaborator**	1	47.32	<0.0001
**Weekday**	6	3.96	0.0006
**Season**	3	1.96	0.1183
**Dam Age**	1	52.54	<0.0001
**Litter Size**[a]	1	17.40	<0.0001
**Litter Size^2**[a]	1	219.15	<0.0001
**Sibling Number**	1	40.91	<0.0001
**Sibling Age**	1	135.48	<0.0001
**Sibling Number*Sibling Age**	1	39.61	<0.0001
**Collaborator*Sibling Age**	1	605.64	<0.0001
**Collaborator*Sibling Number**	1	97.59	<0.0001
**Collaborator*Litter Size**	1	18.72	<0.0001
**Collaborator*Season**	3	3.80	0.0098
**Collaborator*Weekday**	6	7.22	<0.0001

n.a.=not applicable.

[a]Variable Litter Size was centered by its mean.
